# Single-Nucleus Chromatin Accessibility Landscape Reveals Diversity in Regulatory Regions Across Distinct Adult Rat Cortex

**DOI:** 10.3389/fnmol.2021.651355

**Published:** 2021-05-17

**Authors:** Yeya Yu, Xiaoyu Wei, Qiuting Deng, Qing Lan, Yiping Guo, Lei Han, Yue Yuan, Peng Fan, Peiying Wu, Shuncheng Shangguan, Yang Liu, Yiwei Lai, Giacomo Volpe, Miguel A. Esteban, Chuanyu Liu, Yong Hou, Longqi Liu

**Affiliations:** ^1^BGI College, Zhengzhou University, Zhengzhou, China; ^2^BGI-Shenzhen, Shenzhen, China; ^3^BGI Education Center, University of Chinese Academy of Sciences, Shenzhen, China; ^4^CAS Key Laboratory of Regenerative Biology, Guangzhou Institutes of Biomedicine and Health, Chinese Academy of Sciences, Guangzhou, China; ^5^College of Veterinary Medicine, Jilin University, Changchun, China; ^6^Joint School of Life Sciences, Guangzhou Medical University and Guangzhou Institutes of Biomedicine and Health, Chinese Academy of Sciences, Guangzhou, China; ^7^Laboratory of Integrative Biology, Guangzhou Institutes of Biomedicine and Health, Chinese Academy of Sciences, Guangzhou, China; ^8^Bioland Laboratory, Guangzhou Regenerative Medicine and Health Guangdong 16 Laboratory, Guangzhou, China; ^9^Shenzhen Bay Laboratory, Shenzhen, China; ^10^Shenzhen Key Laboratory of Single-Cell Omics, BGI-Shenzhen, Shenzhen, China

**Keywords:** rat cerebral cortex, chromatin accessibility, snATAC-seq, regulatory element, transcription factor

## Abstract

Rats have been widely used as an experimental organism in psychological, pharmacological, and behavioral studies by modeling human diseases such as neurological disorders. It is critical to identify and characterize cell fate determinants and their regulatory mechanisms in single-cell resolutions across rat brain regions. Here, we applied droplet-based single-nucleus assay for transposase-accessible chromatin using sequencing (snATAC-seq) to systematically profile the single-cell chromatin accessibility across four dissected brain areas in adult *Sprague–Dawley* (SD) rats with a total of 59,023 single nuclei and identified 16 distinct cell types. Interestingly, we found that different cortex regions exhibit diversity in both cellular compositions and gene regulatory regions. Several cell-type-specific transcription factors (TFs), including SPI1, KLF4, KLF6, and NEUROD2, have been shown to play important roles during the pathogenesis of various neurological diseases, such as Alzheimer’s disease (AD), astrocytic gliomas, autism spectrum disorder (ASD), and intellectual disabilities. Therefore, our single-nucleus atlas of rat cortex could serve as an invaluable resource for dissecting the regulatory mechanisms underlying diverse cortex cell fates and further revealing the regulatory networks of neuropathogenesis.

## Introduction

The rat, one of the most widely used model organisms, is invaluable for biomedical research by modeling human physiology and diseases (Grigoruţă et al., 2020; Ma et al., 2020). Compared to laboratory mice, rats have a larger body and organ size as, on average, adult rats weigh eight to ten times more than adult mice, making them more suitable for experimental surgeries (Nesbitt, 1974; Atchley et al., 1992; Pehrson et al., 2013; Ellenbroek and Youn, 2016). For example, the larger brain size of rats greatly facilitates sophisticated procedures such as *in vivo* electrophysiology, neurosurgery, and neuroimaging.

Besides the morphological differences, major functional differences are also identified and characterized between rat and mouse brains. For example, there is increasing evidence supporting a critical role of 5-hydroxytrypta-mine-6 (5-HT6) receptors in memory formation and cognitive functions during learning (Lindner et al., 2003). The 5-HT6 receptors are widely expressed in human and rat brains but are not detected in mouse brains (Meneses, 2001; Ohno et al., 2011). Moreover, the hippocampus neurogenesis (the process through which stem cells self-renew and generate new mature neurons) also exhibits a difference between mice and rats. Neural stem cells in the hippocampus are able to proliferate and differentiate into neurons even in adulthood, and this process is thought to be related to learning and memory (Lazarov and Hollands, 2016). Recent studies have shown that the neurogenesis rate in the hippocampus of adult rats is much greater than that of adult mice, revealing differences in the plasticity of neural cells between rats and mice (Snyder et al., 2009).

The rat has become a powerful model organism for studying neurological diseases like Parkinson’s disease (PD; Tiklova et al., 2020), Alzheimer’s disease (AD), astrocytic gliomas, and intellectual disability. The rat’s genomic and transcriptomic information have recently been made available to the community, including genome annotation from the Rat Genome Sequencing Project Consortium (Gibbs et al., 2004) and rat RNA-Seq transcriptomic BodyMap information (Yu et al., 2014). Furthermore, gene-editing technologies, including TALENs, ZFNs, and the CRISPR/Cas system, have been successfully applied to rats (Geurts et al., 2010; Tesson et al., 2011; Shao et al., 2014). With the advancement of genetics and genomics technologies, using a rat as an experimental model, several factors have been identified that play critical roles in both brain physiology and disease (Xu et al., 2019; Berg et al., 2020). To further characterize relationships among these key factors in neurological diseases and identify novel factors, a comprehensive regulatory network of rat brain tissue is of primary importance. An integrative analysis of expression and epigenetic datasets in disease-relevant cell types would be of great potential in dissecting disease pathways and mechanisms.

The cerebral cortex, the brain’s outer layer, is composed of many different regions. These regions are involved in complex brain functions that are not only related to various sensations and movements of the body but also to various cognitive abilities such as language and thinking. Here, we employed the droplet-based high-throughput snATAC-seq to comprehensively profiled the chromatin accessibility status on the rat auditory cortex (AC), primary visual cortex (V1), somatosensory cortex (SC), and motor cortex (MC). We obtained a total of 59,023 cells that passed the quality control (QC) from the rat cortex, creating a new single nucleus database that would serve as a helpful resource for future studies in neurological diseases.

## Materials and Methods

### Tissue Dissection and Preservation

The use of rats in relevant experimental studies was approved by the Institutional Review Board on the Ethics Committee of BGI (Permit No. BGI-IRB A20020). One 7–8-month-old healthy adult female Sprague–Dawley (SD) rat was used in this study and was purchased from Jiangsu Ailingfei Biotechnology Company Limited. The rat was transported by air to the Guangzhou Institute of Biomedicine and Health (GIBH) of the Chinese Academy of Sciences, where GIBH colleagues helped with tissue dissection. Upon arrival at GIBH, the rat was sacrificed by carbon dioxide asphyxiation and proceeded to dissection according to standard protocol (Paxinos and Watson, 2007; Yang et al., 2018). Each tissue type was stored in a cryopreservation tube. After being snap-frozen in liquid nitrogen, the dissected tissue was transported on dry ice to BGI-Shenzhen and was immediately stored in a liquid nitrogen tank upon arrival.

### Nuclei Isolation From Dissected Tissue

**Figure 1:**
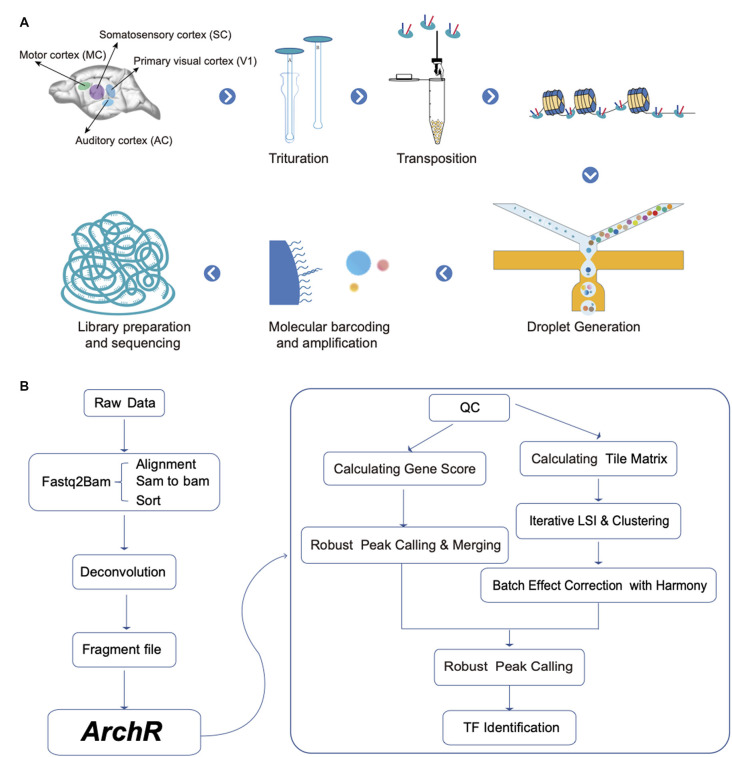
Schematic diagram for experimental and data analysis.** (A)** A total of four different cortex regions from adult rat brains were collected for single-nucleus assay for transposase-accessible chromatin using sequencing (snATAC-seq) profiling. **(B)** The analysis workflow for snATAC-seq profiles.

In this study, we specifically focused on the dissected AC, V1, SC, and MC from rats, as shown in Figure 1A. Nuclei were isolated from the frozen cortex according to an established protocol with minor modifications (Bakken et al., 2018; Thrupp et al., 2020). The whole operation was carried out on ice. Briefly, frozen tissue was cut into pieces and transferred to a 2 mL KIMBLE Dounce tissue grinder (Sigma #D8938-1SET) with 2 mL of ice-cold homogenization buffer [20 mM Tris pH 8.0 (Thermo Fisher Scientific)], 500 mM sucrose (Sigma), 50 mM KCl (Thermo Fisher Scientific), 10 mM MgCl_2_ (Thermo Fisher Scientific), 0.1% NP-40 (Roche), 1× protease inhibitor cocktail (Roche), and 1% nuclease-free BSA, and 0.1 mM DTT). Tissues were homogenized by 15 strokes of the loose dounce pestle, and the homogenate was filtered through a 70 μM cell strainer (Falcon). Next, the filtered homogenate was homogenized by five strokes of the tight pestle to release nuclei, and it was again filtered through a 30 μM cell strainer (Sysmex) into a 15 mL centrifuge tube. Nuclei pellets were then obtained by centrifuging at 500 *g* for 5 min at 4°C. Nuclei were then washed twice with 1 ml of ice-cold blocking buffer (1× PBS supplemented with 1% BSA) followed by another step of centrifugation at 500 g for 5 min at 4°C. Finally, we resuspended the nuclei in 50 μL of 1× PBS containing 1% BSA and counted them with DAPI.

### snATAC-Seq Library Preparation and Sequencing

To prepare single-nucleus ATAC-seq libraries, we used DNBelab C Series Single-Cell ATAC Library Prep Set (MGI, #1000021878; Han et al., 2020). In total, we generated 11 libraries, including two libraries from AC and three libraries each from the other types of cortices. Briefly, transposed single-nucleus suspensions were converted to barcoded snATAC-seq libraries, through procedures including droplet encapsulation, pre-amplification, emulsion breakage, capture beads collection, DNA amplification, and purification. Indexed sequencing libraries were prepared according to the user guide. Concentrations of sequencing libraries were measured with Qubit ssDNA Assay Kit (Thermo Fisher Scientific). All libraries were sequenced using a paired-end 50 sequencing scheme by the BGISEQ-500 platform at China National GeneBank (CNGB; Huang et al., 2017).

### Preprocessing and QC of the snATAC-seq Datasets

Raw sequencing reads from BGISEQ-500 sequencer were filtered and demultiplexed using PISA and aligned to the rat genome (Rnor_6.0^1^). The fragment file of each snATAC-seq library was used for downstream analysis (Figure 1B). The transcription start site (TSS) enrichment score and fragment number of each nucleus were calculated by using ArchR software (Granja et al., 2021). Nuclei with TSS enrichment score less than five and fragment number less than 1,000 were removed. We created an Arrow file to record all relevant information of each sample library such as metadata, and data matrices). Then we calculated the doublet score with *addDoubletScores* function in ArchR package and filtered doublets by *filterDoublets* function with parameter filterRatio = 2.

### snATAC-Seq Latent Semantic Indexing (LSI) Clustering

An snATAC-seq clustering analysis was performed using ArchR software. To cluster our snATAC-seq data, we first identified a robust set of bins followed by iterative LSI method clustering (Granja et al., 2019; Satpathy et al., 2019). Briefly, we created 500 bp windows tiled across the genome and determined whether each cell was accessible within each window. Next, we performed an LSI dimensionality reduction on these windows with *addIterativeLSI* function in ArchR packages followed by Harmony batch correction (Korsunsky et al., 2019). We performed clustering by Seurat’s *FindClusters* function with parameters “reducedDims = ‘IterativeLSI’, method = ‘Seurat’, resolution = 0.8)”

### snATAC-Seq Gene Activity Scores

Gene scores were calculated using ArchR with default parameters (Pliner et al., 2018; Lareau et al., 2019). Briefly, ArchR uses a distance-weighted accessibility model to infer gene activity scores, which can aggregate accessibility signals in genes and local genomic regions. We calculated the gene activity scores by *addGeneScoreMatrix* function of ArchR package with options addGeneScoreMatrix (geneModel = “exp(−abs(x)/5,000) + exp(−1)”, extendUpstream = c(1,000, 1e^+05^), extendDownstream = c(1,000, 1e^+05^), geneUpstream = 5,000, and geneDownstream = 0, tileSize = 500). The gene activity scores are based on the following observations. The accessibility of the entire gene body contributed to the gene scores. For putative distal regulatory elements, we used an exponential weighting function to calculate the score. We added gene boundaries to reduce the contribution of irrelevant regulatory elements to gene scores. The resulting gene activity scores were additionally imputed using MAGIC to reduce noise due to snATAC-seq data sparsity. The different gene scores between each cortex of each cell type were calculated by *getMarkerFeatures* function in ArchR software.

### Identification of Cell Types From snATAC-Seq Data

We used gene activity scores to identified different cell types for various marker genes. Astrocytes (cluster 1) were annotated by accessibility near the *Gfap*, *Slc1a2*, and *Aqp4* genes. Endothelial cells (cluster 2) were annotated by accessibility near the *Cldn5* and *Flt1* genes. Excitatory neurons (clusters 3, 4, 5, 6, and 7) were annotated by accessibility near the *Slc17a7* genes. Inhibitory neurons (cluster 8) were annotated by accessibility near the *Gad1* and *Gad2* genes. PVALB neurons (cluster 14) were annotated by accessibility near the *Pvalb* gene. SST neurons (cluster 15) were annotated by accessibility near the *Sst* gene. VIP neurons (cluster 16) were identified based on accessibility near the *Vip* gene. Meningeal cells (cluster 9) were identified based on accessibility near the *Six1* gene. Microglia (cluster 10) was annotated by accessibility near the *Itgam*, *Adgre1*, *P2ry12*, *Tmem119*, *Tgfb1*, and *Apbb1ip* genes. Oligodendrocyte precursor cells (OPC) (cluster 11) were annotated by accessibility near the *Cspg4* and *Pdgfra* gene. Oligodendrocytes (cluster 12) were annotated by accessibility near the *Opalin*, *Mog*, *Mobp*, *Mbp*, and *Cldn11* genes. Pericytes (cluster 13) were annotated by accessibility near the *Pdgfrb* genes. All neuron subsets were mainly determined primarily as neurons, according to the accessibility near the *Nefh*, *Syt1*, and *Rbfox3* genes, and then subdivided according to the origin and accessibility near the other genes mentioned above.

### snATAC-Seq Peak Calling

For each cell type, peak calling on the Tn5-corrected insertions (each end of the Tn5-corrected fragments) using the MACS2 callpeak command with the parameters (shift = −75, extsize = 150, cutoff = 0.1, additionalParams = “—nomodel —nolambda”). The peak summits were then extended by 250 bp on either side to a final width of 500 bp. Then the marker peaks of each cell type were defined by *getMarkerFeatures* function and the *peak AnnoEnrichment* function was used to enrich the motifs in the significant marker peaks (FDR <= 0.01, Log_2_FC >= 1). The scripts used for analysis are in the supplementary material (Supplementary Files 1–5).

## Result and Discussion

### snATAC-Seq Metrics for Quality Control of Individual Cells

**Figure 2:**
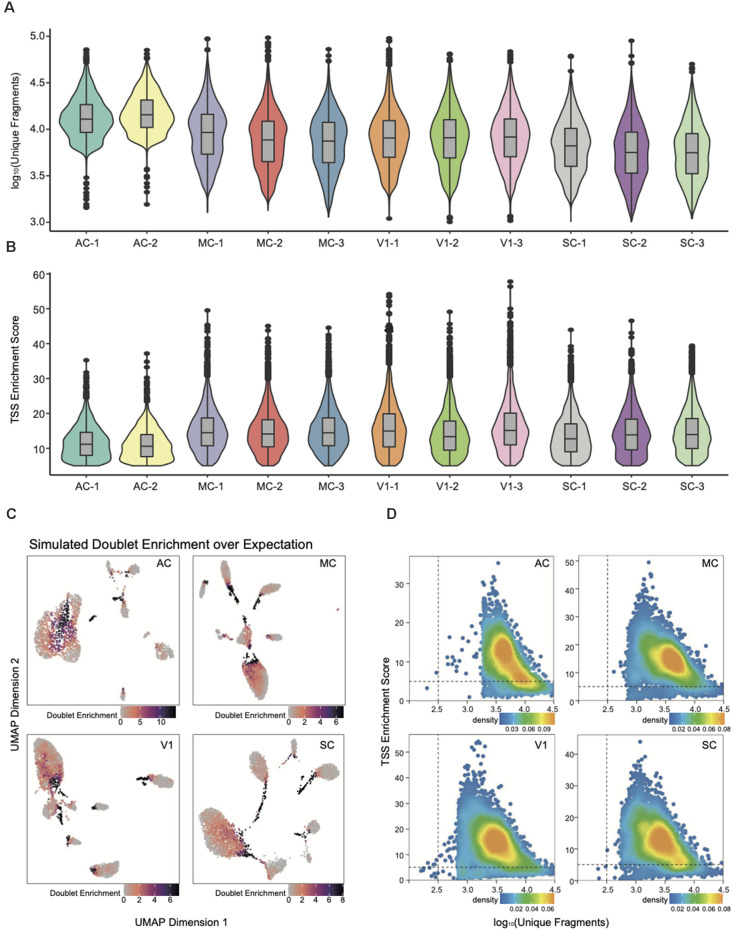
snATAC-seq data quality metrics. **(A)** Violin plot for the number of unique nuclear fragments per library. **(B)** Violin plot for the distribution of transcription start site (TSS) enrichment score per library. **(C)** UMAP of snATAC-seq data showing the simulated doublet enrichment over expectation based on genotyping information. **(D)** Quality control (QC) filtering plots for the auditory cortex (AC), motor cortex (MC), primary visual cortex (V1), and somatosensory cortex (SC) dataset showing the TSS enrichment score vs. unique nuclear fragments of each nucleus.

In this study, we successfully prepared and sequenced 11 snATAC-seq libraries from four distinct rat AC, MC, V1, and SC samples. A total of 6,032 million raw reads were generated, and 59,023 cells were obtained after quality filtering (Supplementary Table 1). Nuclei with fragment numbers less than 1,000 were filtered out, and the median fragments per nucleus was 9,243 (Supplementary Table 1, Figure 2A). First, we evaluated the sequencing data quality for each library through several parameters, including the number of total reads, fraction of read pairs with a valid barcode, Q30 bases in reads, and Q30 bases in barcodes. Around 82.20% of the raw reads were filtered into a total of 4,960 million clean reads. The average of Q30 bases in reads and barcodes is 93.99% and 93.65%, respectively. Next, following data pre-processing *via* alignment, SAM to BAM conversion, and sorting, deconvolution was applied to generate fragment files.

All subsequent QC and downstream analyses were performed with ArchR (Granja et al., 2020). The number of unique fragments and the TSS enrichment score were the most robust metrics for quality control (Fang et al., 2019). The paucity of unique fragments predicts less reliable analysis; the lower TSS enrichment score reflects a lower signal-to-background ratio. The violin plots in Figures 2A,B have demonstrated that each snATAC library is of high data quality by assessing parameters of unique fragment and TSS enrichment. We then integrated the data into four cortex categories, and removed poor quality nuclei with TSS enrichment score less than five and fragment number less than 1,000 for downstream analysis (Supplementary Table 1, Figure 2D).

To further improve the fidelity of downstream data analysis, we performed doublet detection to eliminate the false positives within cell clusters and interconnections among them (Figure 2C). The doublets generation occurred during droplet encapsulation, where more than one cell was captured within a droplet and thus labeled with the same cell barcode. After filtering out doublets with parameter filterRatio = 2, we then could reliably construct the single-nucleus chromatin landscapes of 59,023 for exploration of tissue-specific regulatory elements.

### Cluster Identification and Comparative Analysis Between Regions

**Figure 3:**
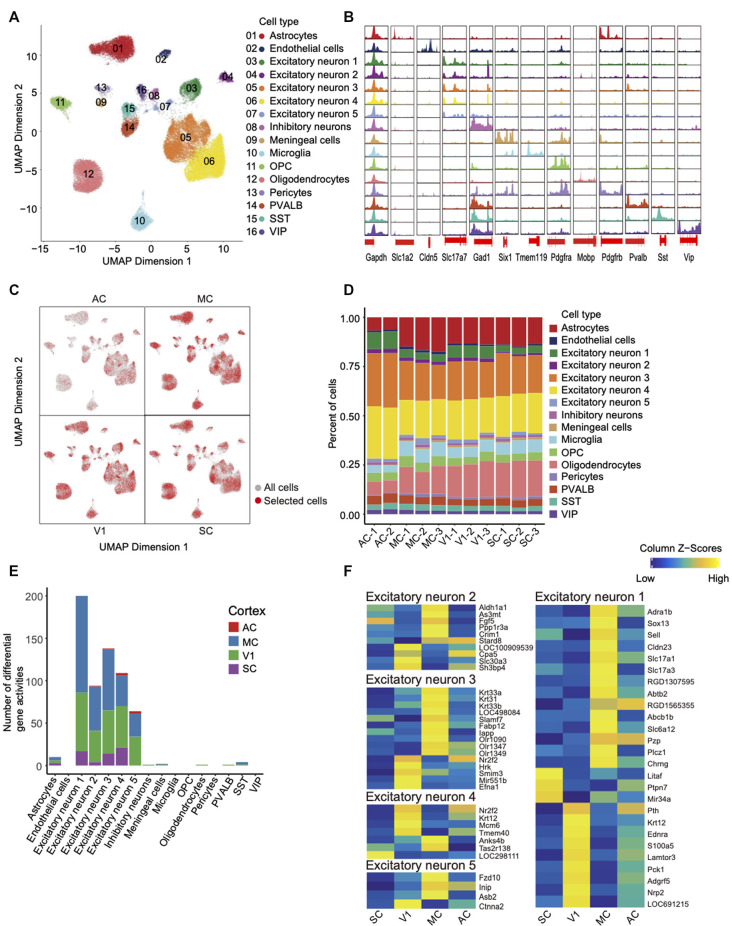
Single-cell chromatin accessibility of rat cortex and inter-regional diversity of excitatory neurons.** (A)** UMAP of snATAC-seq data showing 16 clusters. A total of 59,023 nuclei passed QC and were used for clustering rat cortices into neuronal and non-neuronal cell types. Each cluster is color-coded to distinguish specific cell types. **(B)** Genome browser views of snATAC-seq signal for the indicated housekeeping gene (Gapdh) and cell-type-specific genes. **(C)** UMAP expresses differences in cellular distribution across the four cortex regions, the red dots represent a specific region. **(D)** Histogram shows the number of cell types between each library in different cortices. **(E)** Histogram of the number of different genes between cortical regions within the same cell type. **(F)** Heatmap of gene activity scores for all genes in the four cortices of each excitatory neuron.

We grouped nuclei into 16 distinct clusters by an optimized iterative LSI method (Granja et al., 2019; Satpathy et al., 2019; Figure 3A). Then we annotated each cell cluster by key marker genes that were identified with gene scores (Cusanovich et al., 2018; Pliner et al., 2018; Lareau et al., 2019). The annotated nuclei types were mostly consistent with a previous study focusing on the cerebral cortex, and the number of neuronal nuclei was greater than non-neuronal nuclei (Zhang et al., 2014; Del-Aguila et al., 2019). These 16 cell clusters include nine types of neurons and seven types of non-neurons. These nine types of neurons include five types of excitatory neurons and four types of inhibitory neurons such as PVALB, SST, and VIP. These six types of non-neurons were the astrocytes, endothelial cells, microglia, OPC, oligodendrocytes, pericytes. Detailed information on cell type marker genes was available in Supplementary Figure 1, Supplementary Table 2. We have identified an additional cluster of meningeal cells, which is possibly due to minor sample contamination during tissue dissection.

First, open chromatin peaks were called for each cell-type cluster. We have detected the open chromatin regions around TSSs of key marker genes for each cell type; these findings further validate the quality of our datasets (Figure 3B). For example, peaks were specifically called around the TSSs region of marker gene *Tmem119* in microglia cells but not other cell types. After identifying the cell types, we explored the differences between the four cortex regions. UMAP initially showed that the four cortices share the same cell types but the number of them is different (Figure 3C, Supplementary Figure 2). Second, by comparison of cellular compositions across the four cortex regions, the red dots representing selected cells of each region (Figure 3C). The diversity of cellular compositions across cortex regions was summarized as shown in Figure 3D. We found that excitatory neurons accounted for the largest proportion. The overall proportion is consistent with the proportion of cell types found in the mouse and human brain (Supplementary Figure 3; Cusanovich et al., 2018; Del-Aguila et al., 2019). Third, the gene activity scores were calculated across four cortex regions in each cluster. We identified the differential gene activity with parameters (Log_2_FC > 0.5 and FDR < 0.05), and the results showed that the excitatory neurons owned most of the differential gene activities (Figure 3E, Supplementary Table 3). In Figure 3F, a heat map has illustrated the distinct differential gene activity specifically in each type of excitatory neurons with more stringent parameters (FDR <= 0.01 and Log_2_FC >= 1).

### Inferring Cell Type-Specific TFs

**Figure 4:**
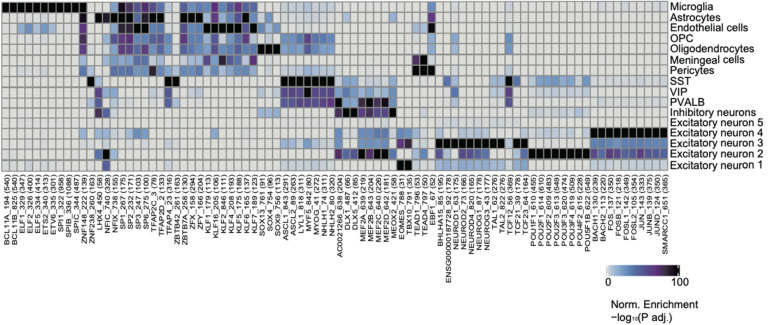
Identification of cell-type-specific chromatin transcription factors (TFs). Heatmap clustering showing the cell-type-specific TFs.

In order to further characterize regulatory mechanisms underlying chromatin landscapes, we used ArchR to identify TFs that are highly correlated with cell type-specific open chromatin regions via unbiased motif finding (Figure 4, Supplementary Table 4). Interestingly, we have identified a group of TFs, which have been shown to be closely related to the pathogenesis and treatment of neurological diseases. The TF PU.1 was a protein encoded by the* Spi1* gene in humans (Ray et al., 1990). PU.1 was highly expressed in the microglia of the brain. Recent evidence from genome-wide association studies suggests that the decrease of PU.1 may limit the neuro-inflammatory response, leading to the delayed onset of AD (Rustenhoven et al., 2018). KLF4, a member of the zinc finger TF family has a great effect on inflammation by mediating endothelial cells. New evidence shows that KLF4 has an important regulatory effect on the neurophysiological and neuropathological processes of AD, suggesting that KLF4 may be a potential therapeutic target for neurodegenerative diseases (Cheng et al., 2018). Glioma is the most common tumor of the central nervous system with an inferior prognosis, and mutations in the *Klf6* gene have been shown to play a role in the pathogenesis of astroglioma (Jeng and Hsu, 2003). It was found that excitatory neuron-specific neurod2 deletion generalizes cellular and behavioral autism spectrum disorder (ASD) phenotypes in full KO mice (Runge et al., 2020). Neurod2 pathogenic mutations cause ASD and intellectual disability.

## Conclusion

Together, in this study, the list of TF candidates identified from cell-type-specific chromatin landscapes would serve as a valuable resource for identifying novel players across distinct cortex regions, and this may further lay the foundation for characterizing the regulatory networks and dissecting mechanisms underlying neurological diseases.

## Data Availability Statement

All raw data have been submitted to the CNGB [Nucleotide Sequence Archive] [https://db.cngb.org/search/project/CNP0001471/] and also the NCBI [SRA] [https://www.ncbi.nlm.nih.gov/search/all/?term=PRJNA684680].

## Ethics Statement

The animal study was reviewed and approved by the Institutional Review Board on Ethics Committee of BGI.

## Author Contributions

YYu, LL, XW and QD conceived the idea. PF and SS collected the samples. YYu and QD generated the data. YYuan and PW assisted with the experiments. XW analyzed the data with the assistance of YYu, YLiu. YYu wrote the manuscript with the input of XW and QL. CL and LL supervised the study and revised the manuscript. LH, YLai and GV provided helpful comments on this study. All authors contributed to the article and approved the submitted version.

## Conflict of Interest

The authors declare that the research was conducted in the absence of any commercial or financial relationships that could be construed as a potential conflict of interest.

## Footnotes

^1^https://github.com/shiquan/PISA

## Supplementary Material

SUPPLEMENTARY FIGURE 1 Cell-type-specific marker genes visualized by UMAP.

SUPPLEMENTARY FIGURE 2 Cluster UMAP of 11 libraries, each color represents a library in four cortical areas.

SUPPLEMENTARY FIGURE 3 Pie chart comparing the proportions of cell types. Refer to the single-cell nuclear transcriptome data of the human cerebral cortex published by Del-Aguila et al. (2019).

SUPPLEMENTARY TABLE 1 snATAC-seq metadata and mapping statistics. *Reads Pass QC: total number of reads minus number of unaligned reads.

SUPPLEMENTARY TABLE 2 Cell type-specific marker genes list. All marker genes used for cell annotation in Figure 3A.

SUPPLEMENTARY TABLE 3 Differential gene activity scores in each cell type.

SUPPLEMENTARY TABLE 4 TF motif enrichment in each cell type.
