# Supplementary material for: Single-Nucleus Chromatin Accessibility Landscape Reveals Diversity in Regulatory Regions Across Distinct Adult Rat Cortex
Source: Front Mol Neurosci. 2021 May 17;14:651355. doi: 10.3389/fnmol.2021.651355 (PMC8166204; doi:10.3389/fnmol.2021.651355)
Supplement: SUPPLEMENTARY FIGURE 1 — Cell-type-specific marker genes visualized by UMAP. [file Data_Sheet_1.ZIP › Supplementary mertials/Supplementary Table 2. Cell type-specific marker genes list.docx]

| Cluster | Cell Type | Gene marker |
| --- | --- | --- |
| 01 | Astrocyte | *Gfap, Slc1a2, Aqp4* |
| 02 | Endothelial cell | *Cldn5, Flt1* |
| 03 | Excitatory neuron 1 | *Slc17a7* |
| 04 | Excitatory neuron 2 | *Slc17a7* |
| 05 | Excitatory neuron 3 | *Slc17a7* |
| 06 | Excitatory neuron 4 | *Slc17a7* |
| 07 | Excitatory neuron 5 | *Slc17a7* |
| 08 | Inhibitory neurons | *Gad1, Gad2* |
| 09 | Meningeal cell | *Six1* |
| 10 | Microglia | *Itgam, Adgre1, P2ry12, Tmem119, Tgfb1 and Apbb1ip* |
| 11 | OPC | *Cspg4,* *Pdgfra* |
| 12 | Oligodendrocytes | *Opalin, Mog, Mobp, Mbp and Cldn11* |
| 13 | Pericytes | *Pdgfrb* |
| 14 | PVALB neurons | *Pvalb* |
| 15 | SST neurons | *Sst* |
| 16 | VIP neurons | *Vip* |
