# Supplementary material for: Single-Nucleus Chromatin Accessibility Landscape Reveals Diversity in Regulatory Regions Across Distinct Adult Rat Cortex
Source: Front Mol Neurosci. 2021 May 17;14:651355. doi: 10.3389/fnmol.2021.651355 (PMC8166204; doi:10.3389/fnmol.2021.651355)
Supplement: SUPPLEMENTARY FIGURE 1 — Cell-type-specific marker genes visualized by UMAP. [file Data_Sheet_1.ZIP › Supplementary mertials/Supplementary Table1. snATAC-seq metadata and mapping statistics.docx]

| **Sample ID** | **Total reads** | **Reads Pass QC** | **Estimated number of cells** | **Median fragments per cell** | **TSS Enrichment** | **Called peak number** |
| --- | --- | --- | --- | --- | --- | --- |
| AC_1 | 559,793,161 | 457,738,428 | 3,270 | 14,552 | 11.92% | 85,777 |
| AC_2 | 610,222,912 | 501,021,345 | 3,493 | 16,280 | 11.53% | 87,928 |
| MC_1 | 577,209,911 | 485,368,120 | 5,232 | 9,865 | 14.86% | 111,154 |
| MC_2 | 560,965,987 | 477,684,632 | 6,991 | 8,383 | 14.34% | 120,418 |
| MC_3 | 498,211,336 | 379,497,584 | 6,546 | 7,950.5 | 14.49% | 117,867 |
| V1_1 | 473,321,713 | 376,715,194 | 5,943 | 8,537 | 14.91% | 98,921 |
| V1_2 | 572,875,403 | 468,769,333 | 5,736 | 8,358.5 | 13.245% | 93,861 |
| V1_3 | 565,755,685 | 477,328,025 | 5,751 | 8,750 | 15.15% | 98,977 |
| SC_1 | 516,077,660 | 405,846,415 | 5,353 | 7,238 | 14.04% | 91,934 |
| SC_2 | 479,233,443 | 405,443,208 | 6,087 | 5,750 | 15.13% | 93,634 |
| SC_3 | 618,719,266 | 531,757,294 | 4,621 | 6,008 | 15.16% | 86,519 |
| Total/Average | 6,032,386,477 | 4,967,169,578 | 59,023 | 9,243 | 14.07% | 1,086,990 |

Table 1. **snATAC-seq metadata and mapping statistics.**
