# Supplementary figures and images for: Single-Nucleus Chromatin Accessibility Landscape Reveals Diversity in Regulatory Regions Across Distinct Adult Rat Cortex
Source: Front Mol Neurosci. 2021 May 17;14:651355. doi: 10.3389/fnmol.2021.651355 (PMC8166204; doi:10.3389/fnmol.2021.651355)

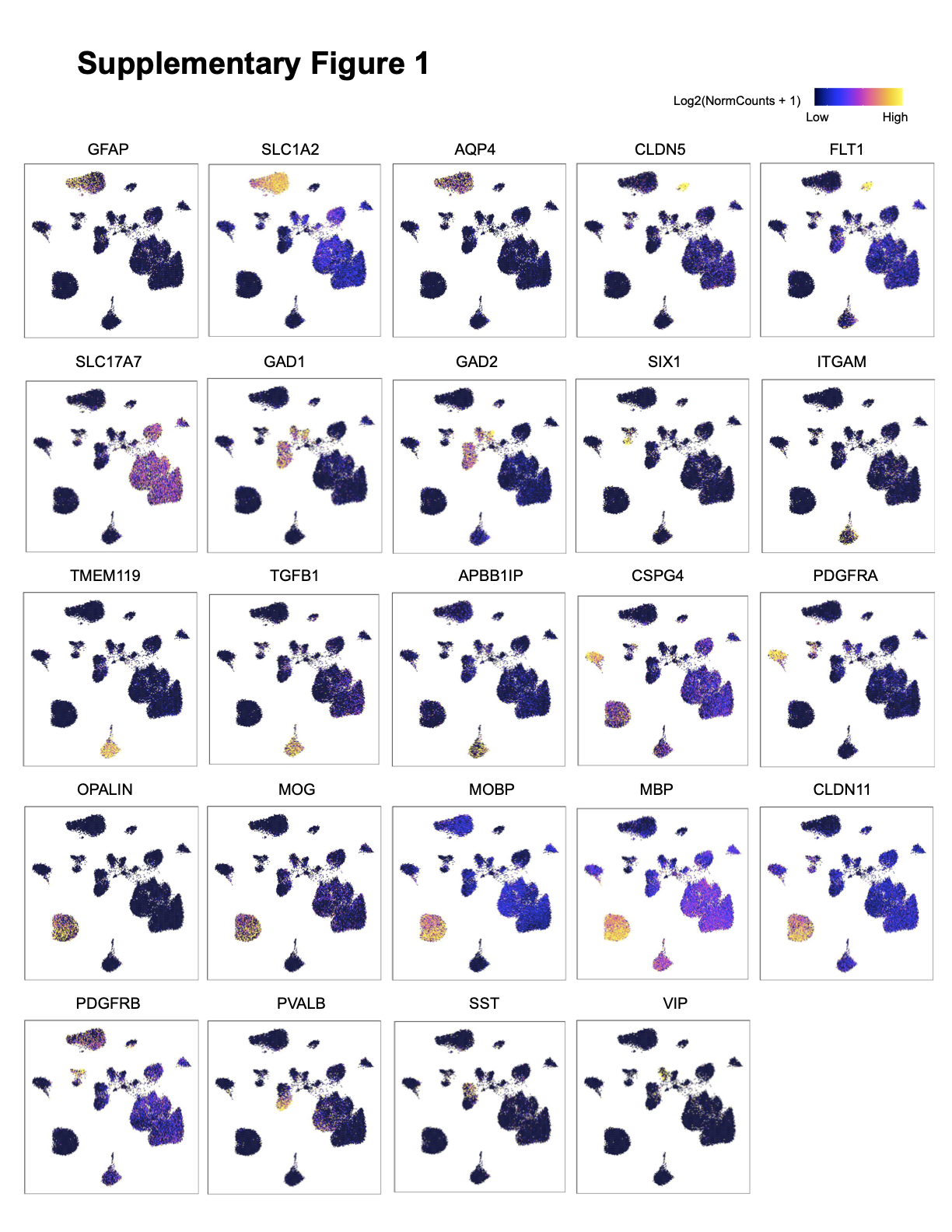

Supplement: SUPPLEMENTARY FIGURE 1 — Cell-type-specific marker genes visualized by UMAP. [file Data_Sheet_1.ZIP › Supplementary mertials/Supplementary Figure 1.tiff]

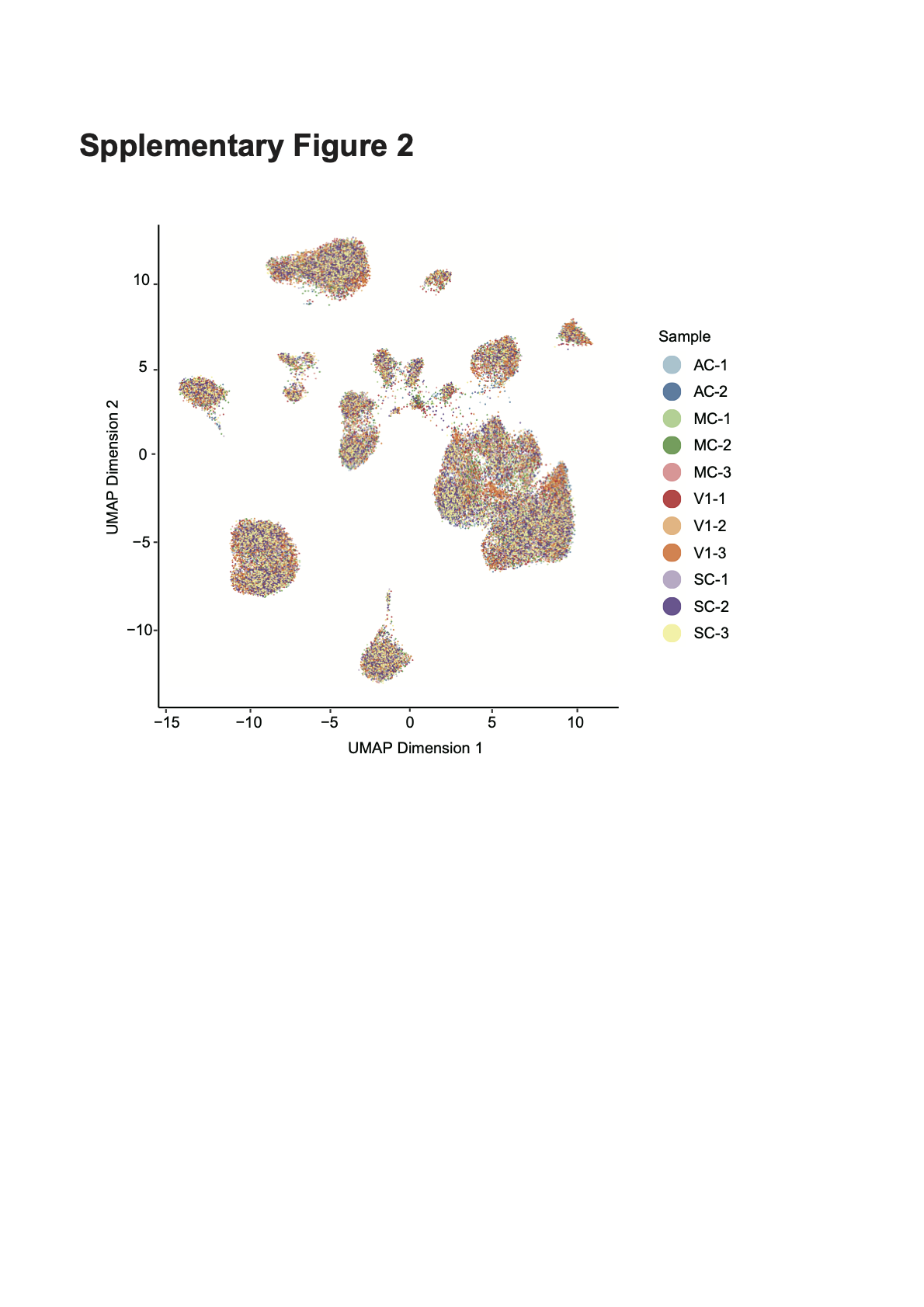

Supplement: SUPPLEMENTARY FIGURE 1 — Cell-type-specific marker genes visualized by UMAP. [file Data_Sheet_1.ZIP › Supplementary mertials/Supplementary Figure 2.tiff]

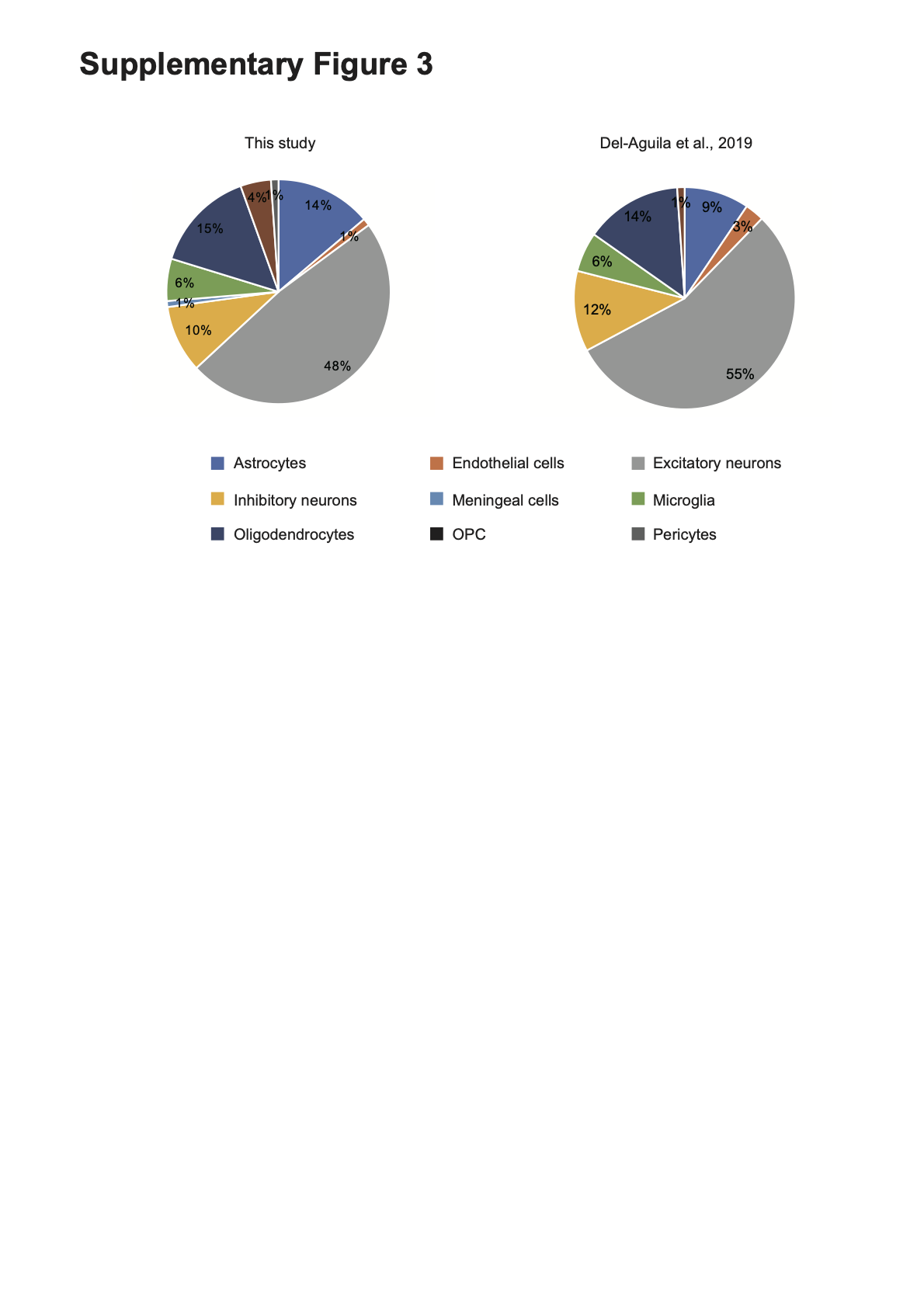

Supplement: SUPPLEMENTARY FIGURE 1 — Cell-type-specific marker genes visualized by UMAP. [file Data_Sheet_1.ZIP › Supplementary mertials/Supplementary Figure 3.tiff]
